# Microstructure and Local Mechanical Properties of the Heat-Affected Zone of a Resistance Spot Welded Medium-Mn Steel

**DOI:** 10.3390/ma14123362

**Published:** 2021-06-17

**Authors:** Manfred Stadler, Ronald Schnitzer, Martin Gruber, Katharina Steineder, Christina Hofer

**Affiliations:** 1 Department of Materials Science, Montanuniversität Leoben, Franz-Josef-Strasse 18, 8700 Leoben, Austria; ronald.schnitzer@unileoben.ac.at (R.S.); christina.hofer@unileoben.ac.at (C.H.); 2 Voestalpine Stahl GmbH, Voestalpine-Strasse 3, 4020 Linz, Austria; martin.gruber@voestalpine.com (M.G.); katharina.steineder@voestalpine.com (K.S.)

**Keywords:** third-generation advanced high strength steels (AHSS), medium-Mn steel, resistance spot welding (RSW), heat-affected zone (HAZ), local mechanical properties

## Abstract

The properties of the heat-affected zone (HAZ) are reported to have a great influence on the mechanical performance of resistance spot welded advanced high strength steels. Therefore, in the present work, the HAZ of a medium-Mn steel is characterized regarding its microstructure and its mechanical properties depending on the distance to the fusion zone (FZ). In order to obtain the local mechanical properties of the HAZ, samples were heat-treated in a joule-heating thermal simulator using different peak temperatures to physically simulate the microstructure of the HAZ. By comparing the microstructure and the hardness of these heat-treated samples and the HAZ, the local peak temperatures within the HAZ could be determined. Subsequently, tensile tests were conducted, and the austenite phase fraction was measured magnetically on the physically simulated HAZ samples in order to determine the local mechanical properties of the HAZ. As verified by energy-dispersive X-ray spectroscopy, peak temperatures above 1200 °C led to a uniform distribution of manganese, resulting in a predominantly martensitic microstructure with high strength and low total elongation after quenching. Below 1100 °C, the diffusion of manganese is restricted, and considerable fractions of austenite remain stable. The austenite fraction increases almost linearly with decreasing peak temperature, which leads to an increase of the total elongation and to a slight decrease in the strength, depending on the distance to the FZ. Temperatures below 700 °C exhibit hardly any effect on the initial microstructure and mechanical properties.

## 1. Introduction

The demands of the automotive industry regarding lightweight construction and simultaneous increase of passenger safety require the application of advanced high-strength steels (AHSS). The research currently focuses on the third generation of these high-performance steels, which are characterized by high strength and acceptable ductility at a significantly lower cost compared to the second generation. This generation consists of quenching and partitioning (Q&P) [1], transformation-induced plasticity (TRIP)-aided bainitic ferrite (TBF) steels [2] and medium-Mn steels [3,4]. First introduced in 1972 by Miller [5], the medium-Mn concept was revived in 2007 by Merwin [6], and since then, research on these steels has intensified again. Their microstructure is composed of a ferritic matrix and a high fraction of austenite of about 30%, which, in contrast to Q&P and TBF steels, is not primarily stabilized by carbon enrichment, but mainly by manganese redistribution during an intercritical heat treatment [5,7]. To achieve this, a manganese content of 4–10% is alloyed. The annealing conditions such as temperature, time and heating and cooling rate play an important role in terms of the austenite quantity and stability [8]. In particular, the annealing temperature has to be chosen carefully to obtain metastable austenite that does not transform into martensite during the final cooling step. The stability of the austenite, which depends among other things on its chemical composition, its grain size and the surrounding matrix, is ultimately responsible for the TRIP effect [9]. Depending on the microstructure prior to the intercritical annealing, the shape of the austenite grains can either be globular or lath-like [10]. In this context, a lath-like austenite is reported to be more stable and offers enhanced mechanical properties [10].

Due to the desired establishment of medium-Mn steels for automotive applications, their workability has to be taken into account. In this context, the resistance spot weldability is of particular importance, as this process is the predominant joining technology in the automotive industry [11]. However, the increased alloying content in third-generation AHSS generally results in a pronounced hardening of the fusion zone (FZ), which reduces their weldability compared to mild steels [12]. Consequently, the modification of the microstructure of the FZ, for example, with a second pulse that acts as an in-process heat treatment, represents a well-documented approach to improve their mechanical performance [13,14,15,16,17,18]. This approach has already been successfully adopted to improve the properties of the medium-Mn steel investigated in the present work [19].

In addition to the FZ, the microstructure of the heat-affected zone (HAZ) also influences the mechanical performance of spot-welded AHSS sheets [12,13,20,21,22]. Generally, the HAZ of AHSS can be subdivided into the upper critical heat-affected zone (UCHAZ), where the temperature during welding exceeds the A_3_ temperature; the intercritical heat-affected zone (ICHAZ), where the peak temperature is between A_3_ and A_1_; and the subcritical heat-affected zone (SCHAZ), where the peak temperature is below A_1_ [23]. The UCHAZ of AHSS usually consists of a fully martensitic microstructure, whose grain size decreases with increasing distance to the FZ because of the decreasing peak temperature [12,13,23,24,25]. Therefore, the UCHAZ can be subdivided into a coarse-grained heat-affected zone (CGHAZ) and a fine-grained heat-affected zone (FGHAZ) [18,25]. Additionally, all of the bainite or martensite-containing AHSS exhibit a significantly softened SCHAZ as a result of tempering effects, which is reported to potentially act as a local necking point and therefore influences the mechanical properties of the welds in a negative way [13,23,26,27]. In these regards, the medium-Mn steels differ significantly from other AHSS. First of all, their SCHAZ cannot soften due to the absence of martensite in the base material (BM). Secondly, due to the stabilization by manganese, some retained austenite may still be present in parts of the UCHAZ [28,29,30]. This is the consequence of the slower diffusion of the substitutionally dissolved alloying element manganese compared to the interstitially dissolved carbon at elevated temperatures [31]. The higher temperature in the CGHAZ leads to a more uniform distribution of manganese, and therefore the fraction of austenite is significantly reduced compared to the FGHAZ [29,30].

The tensile properties of these zones are of great importance regarding the mechanical performance of the welds and have already been investigated in the literature for a number of AHSS [24,26,29,31]. As the CGHAZ offers high strength at low total elongation, it is usually very brittle, which may promote crack propagation through this zone [24,26]. Because of grain refinement, both strength and total elongation increase in the FGHAZ [24]. In contrast, the strength in the ICHAZ and SCHAZ is lower compared to the BM at a high total elongation [24]. Embrittlement of the fully martensitic CGHAZ and associated deterioration of the mechanical performance of the welds is also reported for a medium-Mn steel [29]. In the FGHAZ, both the strength and ductility increase due to fine martensite and the presence of austenite. Therefore, a targeted adjustment of this microstructure at the area where failure occurs is very promising. An approach to achieve remarkable improvement of the ductility of the CGHAZ by in situ post-weld heat treatment was already presented by Park et al. [31] and can be seen as a first step towards targeted HAZ design.

However, since the diffusivity of manganese is very sensitive to temperature, it can be assumed that the manganese distribution and thus the austenite stability varies strongly over the entire HAZ of medium-Mn steels, which may lead to position-dependent mechanical properties. Therefore, the present work aims to investigate the thermal stability of the austenite in the HAZ as a function of the distance to the FZ and determine its influence on the local mechanical properties. While all the available studies [24,26,29,31] rely on a finite element (FE) software, namely SORPAS (Lyngby, Denmark) by SWANTEC, to simulate the thermal cycle during resistance spot welding (RSW), a different approach is presented in this work, which allows the precise replication of the temperature profile and the investigation of the entire HAZ in a small step size based on the actual microstructure. To achieve this, samples were heat-treated on a thermal simulator in order to reproduce the microstructure of the HAZ. For this purpose, sheets were rapidly heated to various peak temperatures quickly quenched to physically simulate the thermal profile in the HAZ during the RSW process. By comparing the microstructure and the hardness of the physically simulated HAZ samples and the HAZ, the local peak temperatures within the HAZ during an RSW cycle could be determined. On the physically simulated HAZ samples, the mechanical properties and the retained austenite were ascertained. Knowledge of the local properties allows targeted HAZ engineering to improve the mechanical properties of the weld in the future.

## 2. Materials and Methods

The investigated medium-Mn steel has a nominal composition of 0.1 C/6.4 Mn/0.6 Si (wt%). The resistance spot welded steel sheets were uncoated and had a thickness of 1.18 mm. RSW was conducted on a MFDC-1000 Hz pedestal-type welding machine from Nimak (Wissen, Germany), which is equipped with an AutoSpatz regulator (Matuschek, Alsdorf, Germany) to deliver constant current. As recommended in VDEh SEP1220-2 [32], F1-16-20-6 electrodes that operated at a clamping force of 4.0 kN were used. The samples were welded for 280 ms at a current of 4.8 kA, representing the current, which is just high enough to produce a weld nugget with a diameter of $4\times \sqrt{t}$, where *t* is the sheet thickness.

The different parts of the HAZ were physically simulated by heat-treating sheets of $150\times 20\times 1.2\;mm^{3}$ on a self-constructed joule-heating thermal simulator. The sheets were heated to different peak temperatures ranging from 1300 to 500 °C in steps of 50 °C, at a rate of about 1300 K/s and then quenched as quickly as possible by means of a water spray. The cooling rate was safely above the critical cooling rate to form martensite. A thermocouple Type K (Ni-CrNi) is welded onto edges in the middle of the sample to facilitate the temperature control. The AC power transformer allows a regulation interval of 20 ms, while the temperature is measured each millisecond. The overall temperature field is homogeneously distributed along the full thickness and width of the sample along 50 mm of length.

**Figure 1:**
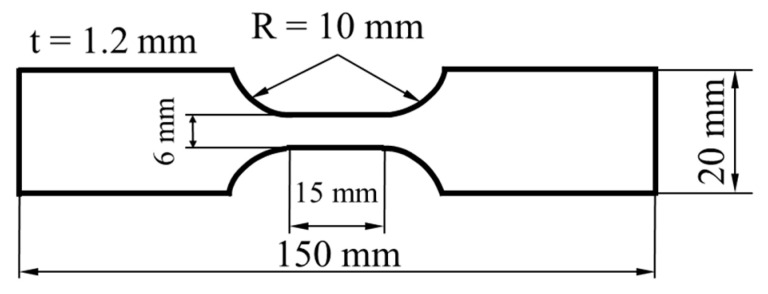
Dimensions of the flat tensile samples.

The mechanical properties of the flat tensile samples (*n* = 1) with the geometry illustrated in Figure 1 were evaluated by tensile testing according to DIN EN ISO 6892-1 [33] on a BETA 400/150-150 testing machine from Messphysik (Fürstenfeld, Austria). The samples were tested longitudinally to the rolling direction. The austenite phase fraction was determined via magnetic saturation measurement (voestalpine-Mechatronics). Hardness testing on the HAZ and the physically simulated HAZ samples was performed at a load of 300 g and a dwell time of 10 s on a Qness hardness tester Q60A+ (Golling, Austria). The hardness of the HAZ was determined from the average of three tests, along vertical lines at an angle of 10° to the sheet/sheet interface according to the isotherms. The vertical distance between the indents within one line was 200 µm. In order to meet the required minimum distances between the indent at a still high small step size of 75 µm, an alternating offset of 100 µm was selected between the respective lines. The hardness of the physically simulated HAZ samples represents an average of five tests.

For the microstructural characterization, cross-sections were ground, polished and etched with a 3% Nital solution. An M1M Imager equipped with an AxioCam MRc5 camera, both from Zeiss (Oberkochen, Germany), was used for light optical microscopy (LOM). The microstructure of the HAZ was determined at eight positions with a distance of 150 µm. Secondary electron (SE) scanning electron microscopy (SEM) images were obtained on a VERSA 3D from FEI (Hillsboro, OR, USA). Electron backscattered diffraction (EBSD) measurements were performed with a step size of 25 nm at a working distance of 15 mm and at an acceleration voltage of 20 kV on the aforementioned SEM, which is equipped with an EDAX Hikari XP (Mahwah, NJ, USA) EBSD system. Since EBSD requires a deformation-free surface, the polished cross-sections were additionally ion milled for 15 min with an ArBlade5000 ion milling system from Hitachi (Chiyoda, Tokio, Japan). Data evaluation was done with the software package OIM Analysis 7 from EDAX (Mahwah, NJ, USA) without any clean-ups. The backscattered electron (BSE) SEM and the energy-dispersive X-ray diffraction (EDX) measurements were conducted on a TESCAN CLARA (Brünn, Czech Republik). EDX measurements were performed at an acceleration voltage of 15 kV, using an X-Max system and the Aztec software from Oxford Instruments (Abington, UK).

## 3. Results and Discussion

### 3.1. Microstructure of the Base Material

**Figure 2:**
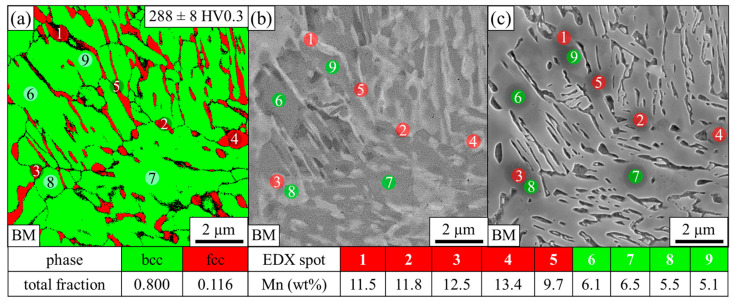
(**a**) EBSD phase map of the base material, (**b**) corresponding BSE image and (**c**) corresponding SE image after Nital etching. The numbered circles (1–9) indicate the points where the manganese content was determined with EDX.

The microstructure of the BM consists of a ferritic (body-centered cubic, bcc) matrix with a considerable fraction of finely dispersed austenite (face-centered cubic, fcc), which is predominantly present in a lath-like arrangement, as shown in the EBSD phase map in Figure 2a, and has a hardness of 288 HV. The data points with a confidence index (CI) below 0.1 were disregarded and are shown in black. They represent either grain boundaries or austenite, which is too fine to be reliably indexed. The numbered circles mark the nine locations where EDX measurements were conducted to determine the manganese content. Due to the similar atomic number of iron and manganese, there is no pronounced chemical contrast using BSE, but the austenite appears slightly brighter due to the higher packing density [34], as can be seen in Figure 2b. Larger, globular austenitic regions contain a manganese content of 11.5 to 12.5 wt% (spots 1–3). However, since the grain size of the austenite is very small, the EDX spot always excites a certain amount of ferrite as well, which generally leads to an underestimation of the manganese content of the austenite. For this reason, more manganese is measured in large austenite islands (spot 4), while less is detected in thin laths (spot 5). The ferritic matrix (spots 6–9) is slightly depleted in manganese compared to the nominal composition of 6.4 wt%. Ferrite that is far from manganese-rich austenite, such as spots 6 and 7, has a higher manganese content than that located adjacent to austenite, such as spots 8 and 9. However, as stated above, the EDX spot excites a relatively large volume that likely contains some austenite, which leads to an overestimation of the manganese content of the ferrite. Due to the higher content of less noble manganese, the austenitic areas were more severely attacked by the Nital etchant compared to the ferritic matrix, as illustrated in the corresponding SE image in Figure 2c. The circular shading at positions 1–9 is caused by carbon contamination during the EDX measurements. At these points, the surface was protected from the etchant; hence, there is no etching attack.

### 3.2. Characterization of the Heat-Affected Zone

**Figure 3:**
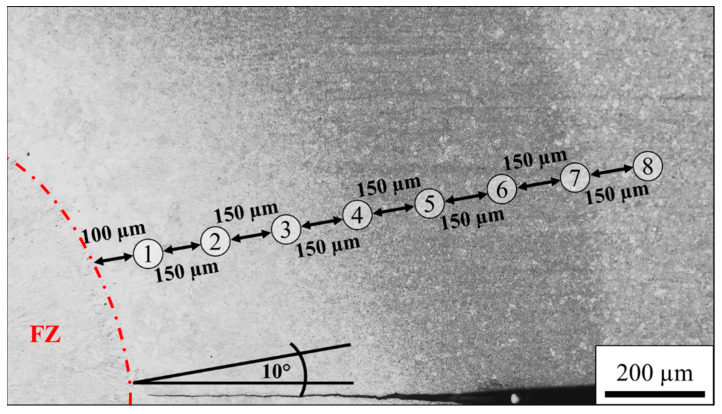
LOM image of the etched HAZ with the different positions numbered from 1–8.

The microstructure of the HAZ was determined at eight positions with a distance of 150 µm, as shown in the LOM image in Figure 3. Nital etching reveals a change in the microstructure between positions 3 and 4 as well as near position 7.

**Figure 4:**
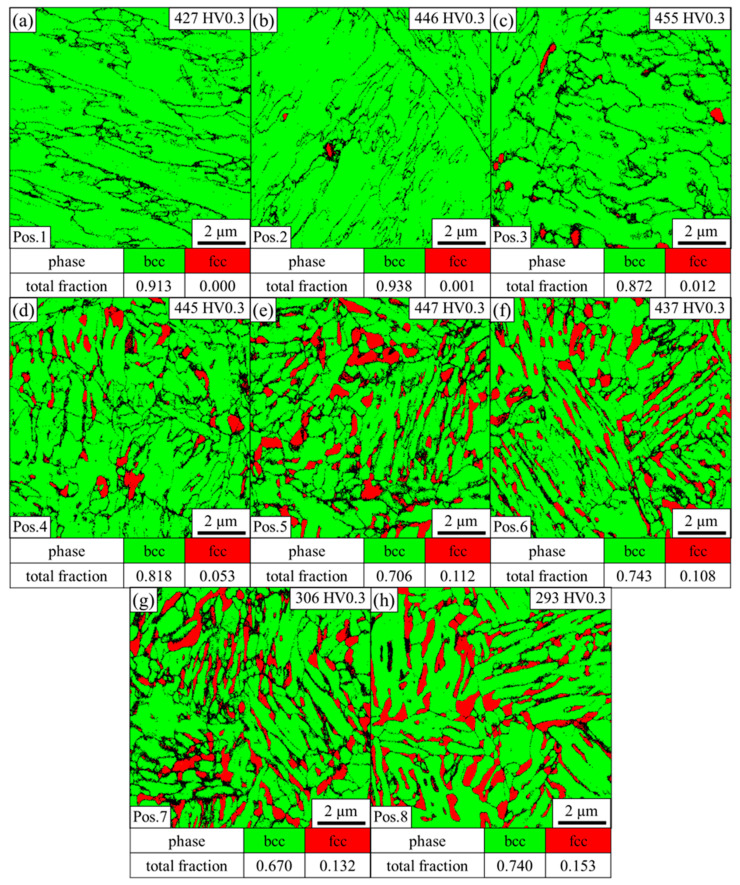
EBSD phase map of the HAZ taken at (**a**) position 1, (**b**) position 2, (**c**) position 3, (**d**) position 4, (**e**) position 5, (**f**) position 6, (**g**) position 7 and (**h**) position 8, as indicated in Figure 3.

As can be seen in the phase map in Figure 4a, no austenite was detected by EBSD at position 1, and the high hardness of 427 HV suggests that the bcc phase represents martensite instead of ferrite. The non-indexed black areas represent martensite block boundaries. The microstructure at position 2 is predominately martensitic with some small individual austenitic regions according to the EBSD phase map illustrated in Figure 4b. The hardness increases to 446 HV. As can be seen in Figure 4c, distinctive austenite grains are detected at position 3. The indexed austenite fraction is still low at 1.2%. However, thin films are below the resolution limit, and therefore it is reasonable to assume that the austenite fraction is underestimated as austenite most likely represents a significant amount of the unindexed points. The hardness further slightly increases to 455 HV. The continuous increase in hardness in this predominantly martensitic region can be explained by grain refinement of the martensite due to the lower austenitization temperature with increasing distance to the FZ. At position 4 (Figure 4d), a considerable amount of austenite of 5.3% with a CI > 0.1 is present. Some of it exhibits a lath-like morphology. The increased austenite fraction leads to a more selective etching attack, which explains the different appearance of this zone between positions 4–6 in the LOM image in Figure 3. The EBSD phase map at position 5 in Figure 4e reveals an austenite phase fraction of 11.2%, which remains almost constant at position 6 (10.8%), as indicated by Figure 4f. The hardness at positions 4–6 is 445, 447 and 437 HV, respectively. Near position 7, the microstructure changes again, as illustrated in Figure 3. This correlates to the ICHAZ, where the material is partly austenitized. Therefore, the microstructure consists of ferrite, martensite and austenite. At position 7, shown in Figure 4g, the austenite fraction increases to 13.2%, and the low hardness of 306 HV illustrates that the bcc phase predominantly represents ferrite instead of martensite. However, the slightly increased hardness compared to the BM indicates that some martensite may be present, which cannot be distinguished from ferrite by EBSD phase maps due to the same crystal structure. A distinction via the image quality as applied in other third-generation AHSS [35,36] is not possible because the low carbon content in the medium-Mn steel results in low distortion of the martensitic lattice. For positions beyond, the A_1_ temperature is no longer exceeded. Therefore, the bcc phase consistently represents the soft ferrite. With a fraction of 15.3%, as illustrated in Figure 4h, the austenite fraction reaches a maximum at position 8 and the hardness of 293 HV hardly differs from that of the BM.

**Figure 5:**
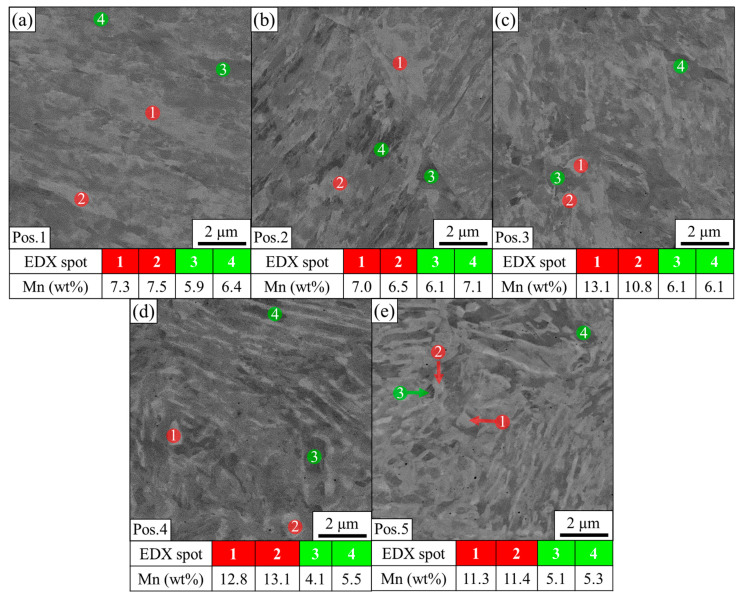
BSE images of the HAZ taken at (**a**) position 1, (**b**) position 2, (**c**) position 3, (**d**) position 4, and (**e**) position 5, as shown in Figure 3. The numbered circles (1–4) indicate the points where the manganese content was determined with EDX for each position.

The increasing fraction of austenite with increasing distance to the FZ is also reflected in BSE images and EDX measurements in Figure 5. As can be seen in Figure 5a,b, the BSE contrast at positions 1 and 2 is very low and mainly caused by the orientation contrast of the martensitic laths. Neither EDX measurements at bright areas (red points), nor at dark areas (green points), indicate pronounced manganese enrichment or depletion, which can be explained by the enabled diffusion of manganese at high temperatures. The BSE contrast at position 3 in Figure 5c is enhanced compared to positions 1 and 2. Analysis of two particularly bright areas (points 1 and 2) reveal a manganese content of 13.1 and 10.8 wt%, respectively. The high manganese content suggests that these areas most likely represent austenite. At position 4 (Figure 5d), manganese is not completely homogenized, and lath-like features resembling the morphology of austenite in the BM can be seen in BSE contrast. Particularly large bright areas have a manganese content of 12.8 and 13.1%, respectively, and therefore represent austenite, while the dark areas are depleted in manganese and correspond to martensite. Position 5 only shows low temperatures, and therefore the characteristic lath-like morphology of the austenite is almost completely retained, resulting in a high contrast in the BSE image in Figure 5e. The bright areas have a manganese content of 11.3 and 11.4%, respectively, while the dark areas are slightly depleted in manganese with 5.1 and 5.3%, respectively.

### 3.3. Characterization of the Samples of the Physically Simulated Heat-Affected Zone

**Figure 6:**
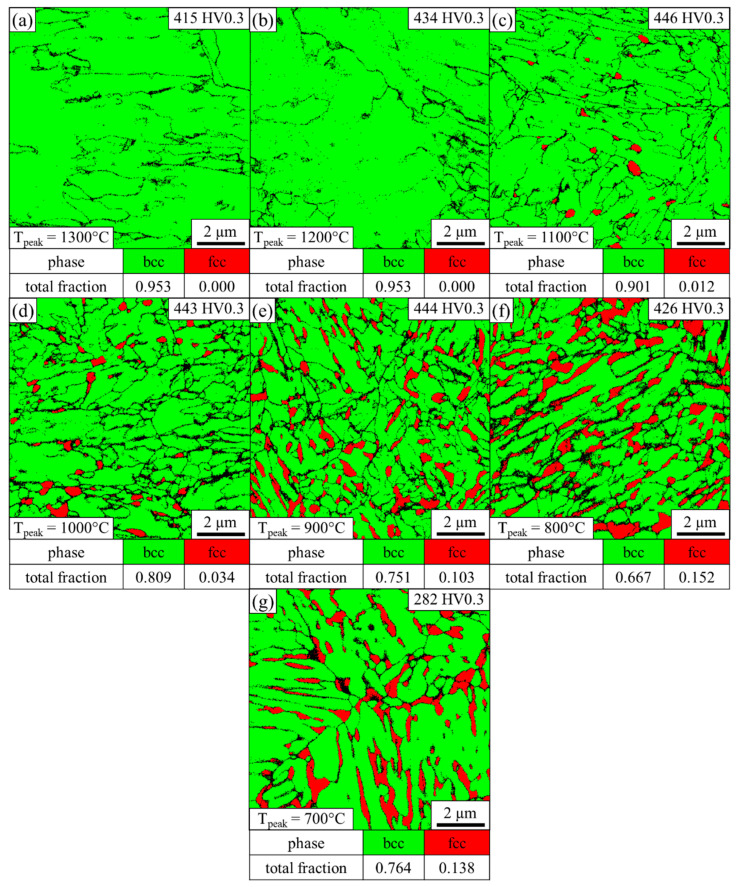
EBSD phase map of the physically simulated HAZ samples heated to a peak temperature of (**a**) 1300 °C, (**b**) 1200 °C, (**c**) 1100 °C, (**d**) 1000 °C, (**e**) 900 °C, (**f**) 800 °C and (**g**) 700 °C and subsequently quenched as quickly as possible.

To simulate the temperature profile in the HAZ during RSW, sheets were heated to different peak temperatures as quickly as possible and then rapidly quenched. In order to correlate the microstructure of the physically simulated HAZ samples to the HAZ, their microstructure was investigated by means of EBSD as well. As can be seen in Figure 6a,b, peak temperatures of 1300 °C and 1200 °C lead to a fully martensitic microstructure after quenching with a hardness of 415 and 434 HV, respectively. At a peak temperature of 1100 °C, a low fraction of 1.2% austenite with a CI > 0.1 is present, as shown in Figure 6c, and the hardness slightly increases to 446 HV. The austenite fraction increases to 3.4% at a peak temperature of 1000 °C with a hardness of 443 HV (Figure 6d). The EBSD phase map at 900 °C (Figure 6e) reveals an austenite fraction of 10.3%, which predominantly exhibits a lath-like morphology. With 444 HV, the hardness stays almost constant. At 15.2%, the austenite fraction detected by EBSD reaches a maximum at a peak temperature of 800 °C, as illustrated in Figure 6f. Since the hardness only moderately decreases to 426 HV, the bcc phase presumably predominantly represents martensite. As can be seen in Figure 6g, a peak temperature of 700 °C results in a low hardness of 282 HV with an austenite fraction of 13.8%.

### 3.4. Correlation of the Physically Simulated HAZ Samples to the Heat-Affected Zone

**Figure 7:**
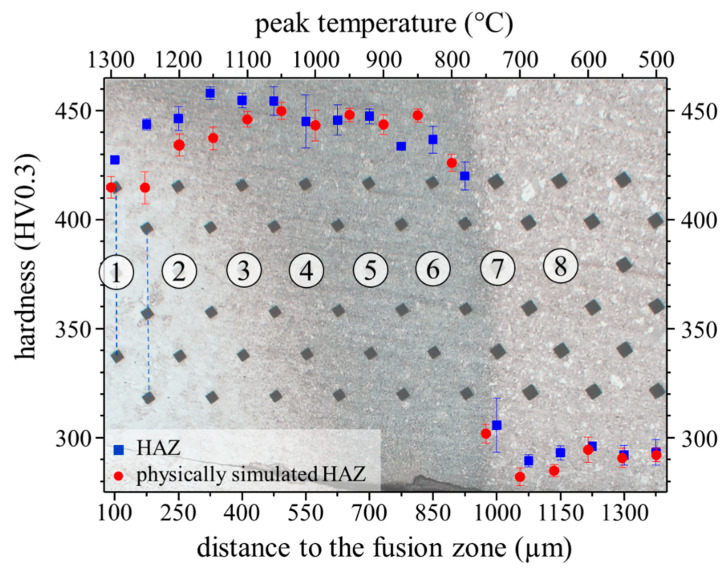
Correlation of the peak temperature of physically simulated HAZ samples to the HAZ, which is shown in the background, by means of the EBSD phase maps and the hardness profiles. The numbered circles (1–8) represent the different positions of the HAZ where the microstructural characterization was done, as already described by Figure 3. The blue squares represent the hardness of the HAZ as a function of the distance to the FZ (lower axis). The red circles represent the hardness of the physically simulated HAZ as a function of the peak temperature (upper axis).

In order to determine the local peak temperatures that were present in the HAZ during welding, the microstructure and the hardness of the physically simulated HAZ samples were compared to the HAZ. The EBSD phase maps of the physically simulated HAZ samples show that the microstructure at the peak temperatures of 1300 °C and 1200 °C is fully martensitic. A minor fraction of 1.2% austenite is detected at a peak temperature of 1100 °C, which corresponds to position 3 in the HAZ. As illustrated in Figure 4c, position 3 represents the closest point to the FZ where individual austenitic grains and a comparable fraction of austenite were detected by EBSD. The ICHAZ represents another characteristic point of reference, since it is accompanied by a sharp drop in hardness due to the change of a martensitic to a ferritic matrix. From the optical micrograph in Figure 3 and the sudden decrease in hardness from 437 HV at position 6 (Figure 4f) to 306 HV at position 7 (Figure 4g), it can be inferred that the ICHAZ is close to position 7. In the physically simulated HAZ samples, the hardness abruptly drops from 426 HV at a peak temperature of 800 °C (Figure 6f) to 282 HV at a peak temperature of 700 °C (Figure 6g). Thus, it can be concluded that the ICHAZ has a temperature between 700 and 800 °C during the RSW cycle. The remaining points were fitted under the assumption of a roughly linear decrease of the peak temperature with increasing distance to the FZ, as can be derived from the results of Park et al. [30]. As shown by Figure 7, the resultant hardness profile of the HAZ (blue squares) and the physically simulated HAZ samples (red circles) show good agreement. Further, the austenite phase fraction and morphology in the EBSD phase maps of the HAZ in Figure 4 and the physically simulated HAZ samples in Figure 6 also correspond well. In summary, the temperature profile of the HAZ could be reproduced and therefore enable the determination of the austenite fraction and the local mechanical properties of the HAZ by means of the physically simulated HAZ samples.

### 3.5. Austenite Fraction and Local Mechanical Properties

**Figure 8:**
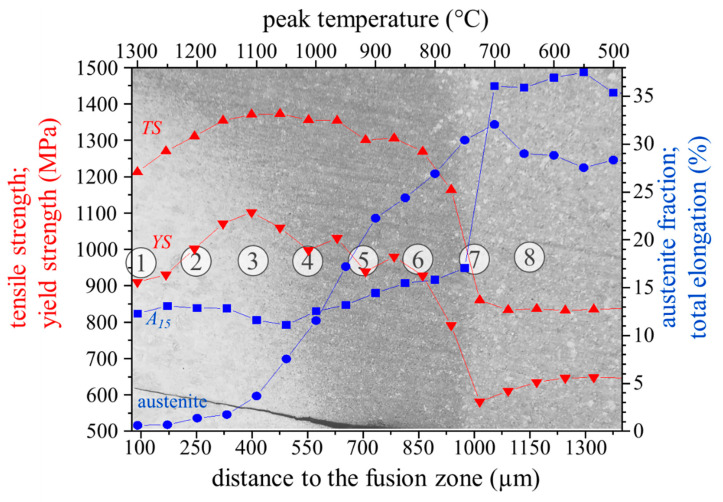
Austenite fraction (blue circles), tensile strength TS (upwards directed red triangle), yield strength YS (downwards directed red triangle) and total elongation A_15_ (blue squares) of the simulated HAZ. The microstructure of the HAZ after Nital etching is shown in the background. The numbered circles (1–8) represent the different positions of the HAZ where the microstructural characterization was done, as already described by Figure 3.

Although EBSD phase maps allow a qualitative comparison of the austenite fraction, as demonstrated in the previous sections, the extremely fine microstructure results in a systematic underestimation of the austenite fraction by EBSD. Therefore, it was quantified on the physically simulated HAZ samples using magnetic saturation measurements. The magnetic determination of the austenite fraction of the BM revealed a fraction of 27.2%, which illustrates on the one hand the considerable underestimation by EBSD (11.6%) and on the other hand shows that the medium-Mn steel investigated in the present work has a significantly higher austenite fraction than medium-Mn steels in comparable studies [28,29]. The yield strength (YS) of the BM is 639 MPa, the tensile strength (TS) is 864 MPa and the total elongation (A_15_) is 33.6%, as determined via tensile testing of a flat tensile sample. Since the peak temperatures of the physically simulated HAZ samples were correlated to the thermal profile of the HAZ, as described in the previous section, the austenite fraction and the local mechanical properties of the HAZ can be determined and are summarized in Figure 8. The results show that the austenite fraction is close to zero above a peak temperature of 1150 °C. With this predominantly martensitic phase, both YS and TS increase continuously with greater distance to the FZ. This is due to decreasing grain size at lower peak temperatures, and a maximum of 1102 MPa and 1371 MPa is reached near position 3. A_15_ remains at a more or less constant level between 11.6% and 13.1%. The austenite fraction increases almost linearly from 1.7% at position 3 to 26.9% at position 6. The increasing fraction of austenite leads to a moderate increase in A_15_ from 11.6% to 15.8% in this zone. YS and TS are determined by the interplay of the strength-increasing mechanism of grain refinement and the strength-decreasing effect of the reduced martensite phase fraction. Both decrease continuously and reach a value of 928 MPa and 1269 MPa, respectively, in the very outer UCHAZ.

In the very narrow ICHAZ, which has a peak temperature of approximately 750 °C, the ferrite is only partly transformed into austenite during heating, and the final microstructure after quenching consequently consists of a mixture of austenite, ferrite and some martensite. This leads to a significant reduction in YS and TS to 791 MPa and 1164 MPa, respectively, with a simultaneous still slight increase of A_15_ to 17.1%. At a peak temperature of 700 °C, no martensite is present, and therefore both YS and TS decrease tremendously to 581 MPa and 861 MPa, respectively, and A_15_ increases sharply to 36.1%. With 32.1%, the austenite fraction reaches a maximum at this position and is noticeably higher compared to the BM. This may be because at this temperature, small amounts of ferrite are still transformed into austenite during heating. In this temperature regime, manganese enrichment may take place, which stabilizes the newly formed austenite. Therefore, no transformation into martensite during the final quenching takes place, and more retained austenite is stable. Lower temperatures do not influence the microstructure, and therefore the austenite fraction and the mechanical performance are equal to those of BM.

To sum up, the austenite retention and local mechanical properties of the HAZ strongly depend on the distance to the FZ, which illustrates the importance of investigating it with a small step size. It is shown that the outer FGHAZ with a high phase fraction of austenite does not display the highest strength, due to the grain refinement of the martensite as suggested in the literature [29], but the zone near position 3 does, where little austenite is present. With increasing distance to the FZ, the strength of the UCHAZ is determined by the interplay of the strength-increasing mechanism of grain refinement and the strength-decreasing effect of the reduced martensite phase fraction, while elongation continuously increases. Nevertheless, it must be mentioned that the mechanical properties of the physically simulated HAZ samples may not exactly represent the local mechanical properties of the HAZ, because, for example, differences in thermal history including holding time may affect the yield stress and plastic flow behavior.

## 4. Summary

In the present work, the microstructure and local mechanical properties of the HAZ of a resistance spot welded medium-Mn steel were investigated. The presented approach provides a way to obtain the temperature profile of the HAZ with a small step size and without relying on complex FE simulations and the most accurate data input data derived from time- and money-consuming investigations. For this purpose, the entire HAZ was characterized by means of SEM, EBSD and hardness measurements and compared to samples heat-treated on a thermal simulator. The temperature profile of the HAZ could be reproduced, and therefore the determination of the austenite phase fraction and the local mechanical properties of the HAZ was possible. Based on these findings, it can be concluded that the austenite in medium-Mn steels exhibits high thermal stability, which leads to highly position-dependent mechanical properties. It is shown that peak temperatures above 1200 °C lead to a uniform distribution of manganese, resulting in a predominantly martensitic microstructure with high strength and low total elongation after quenching. Below 1100 °C, the diffusion of manganese is inhibited, and a considerable fraction of austenite remains stable. The austenite fraction increases almost linearly with decreasing temperature, which leads to an increase in the total elongation and a slight decrease in the strength with a greater distance to the FZ. Temperatures below 700 °C exhibit hardly any effect on the microstructure and mechanical properties. The microstructural and mechanical characterization conducted in this work, combined with the simulated temperature profile, is intended to be the basis for future efforts to specifically manipulate the microstructure of the HAZ, for example, by means of a post-weld heat treatment, in order to potentially improve the mechanical performance of the entire weld.

## Data Availability

Not applicable.
